# Flavoring Chemicals in E-Cigarettes: Diacetyl, 2,3-Pentanedione, and Acetoin in a Sample of 51 Products, Including Fruit-, Candy-, and Cocktail-Flavored E-Cigarettes

**DOI:** 10.1289/ehp.1510185

**Published:** 2015-12-08

**Authors:** Joseph G. Allen, Skye S. Flanigan, Mallory LeBlanc, Jose Vallarino, Piers MacNaughton, James H. Stewart, David C. Christiani

**Affiliations:** Harvard T.H. Chan School of Public Health, Boston, Massachusetts, USA

## Abstract

**Background:**

There are > 7,000 e-cigarette flavors currently marketed. Flavoring chemicals gained notoriety in the early 2000s when inhalation exposure of the flavoring chemical diacetyl was found to be associated with a disease that became known as “popcorn lung.” There has been limited research on flavoring chemicals in e-cigarettes.

**Objective:**

We aimed to determine if the flavoring chemical diacetyl and two other high-priority flavoring chemicals, 2,3-pentanedione and acetoin, are present in a convenience sample of flavored e-cigarettes.

**Methods:**

We selected 51 types of flavored e-cigarettes sold by leading e-cigarette brands and flavors we deemed were appealing to youth. E-cigarette contents were fully discharged and the air stream was captured and analyzed for total mass of diacetyl, 2,3-pentanedione, and acetoin, according to OSHA method 1012.

**Results:**

At least one flavoring chemical was detected in 47 of 51 unique flavors tested. Diacetyl was detected above the laboratory limit of detection in 39 of the 51 flavors tested, ranging from below the limit of quantification to 239 μg/e-cigarette. 2,3-Pentanedione and acetoin were detected in 23 and 46 of the 51 flavors tested at concentrations up to 64 and 529 μg/e-cigarette, respectively.

**Conclusion:**

Because of the associations between diacetyl and bronchiolitis obliterans and other severe respiratory diseases observed in workers, urgent action is recommended to further evaluate this potentially widespread exposure via flavored e-cigarettes.

**Citation:**

Allen JG, Flanigan SS, LeBlanc M, Vallarino J, MacNaughton P, Stewart JH, Christiani DC. 2016. Flavoring chemicals in e-cigarettes: diacetyl, 2,3-pentanedione, and acetoin in a sample of 51 products, including fruit-, candy-, and cocktail-flavored e-cigarettes. Environ Health Perspect 124:733–739; http://dx.doi.org/10.1289/ehp.1510185

## Introduction

The World Health Organization (WHO) reports that $3 billion was spent on electronic cigarettes (e-cigarettes) in 2013 in the United States alone, with sales expected to increase 17-fold in 15 years. The CDC (Centers for Disease Control and Prevention) estimates that 1.78 million children tried e-cigarettes as of 2012, with 160,000 of them reporting that they had not used tobacco cigarettes (CDC 2013). E-cigarettes are not currently regulated; the U.S. Food and Drug Administration (FDA), which has the authority to regulate certain tobacco and nicotine-containing products under the Family Smoking Prevention and Tobacco Control Act (2009) has issued a proposed rule to include e-cigarettes under this Act (FDA 2014). Although the popularity and use of e-cigarettes continues to increase, data are lacking on the exposures and potential human health effects of the use of e-cigarettes.

Concerns regarding e-cigarettes primarily focus on nicotine exposure, secondhand exposure, the potential for e-cigarettes to be a gateway to cigarette use, and renormalization/social acceptance of smoking (Bell and Keane 2014; Coleman et al. 2014; Goniewicz et al. 2014a; Long 2014; McMillen et al. 2015; Trehy et al. 2011). Other recent investigations have focused on the chemical content of the e-cigarettes beyond nicotine, with researchers finding that users of e-cigarettes are exposed to carbonyl compounds, aldehydes, fine particulate matter, metals, propylene glycol, glycerol, formaldehyde, VOCs, and other additives (Bekki et al. 2014; Callahan-Lyon 2014; Cheng 2014; Goniewicz et al. 2014b; Hutzler et al. 2014; Jensen et al. 2015; Orr 2014; Pellegrino et al. 2012; Uchiyama et al. 2013; Williams et al. 2013). However, despite > 7,000 flavors of e-cigarettes currently marketed (Zhu et al. 2014), only three papers have been published that focus on exposure to flavoring chemicals specifically (Farsalinos et al. 2015; Hutzler et al. 2014; Behar et al. 2014), and one opinion piece in *JAMA* that highlights the potential respiratory health effects from using flavored e-cigarettes (Barrington-Trimis et al. 2014).

The use of flavorings in food products gained public attention in the early 2000s because of reports of serious lung disease in microwave popcorn workers (Hilts 2001). The flavoring chemicals involved were on the Generally Recognized As Safe (GRAS) list that applies only to ingestion, but exposures were occurring via inhalation and very little was known about potential inhalation hazards of these chemicals at that time (FDA 2015). In May 2000, eight persons who had previously worked at a microwave popcorn–processing plant were reported to have severe bronchiolitis obliterans (Kreiss et al. 2002), an irreversible loss of pulmonary function that can become so severe that the only treatment option may be a lung transplant (OSHA 2007). Researchers from the National Institute of Occupational Safety and Health (NIOSH) Division of Respiratory Disease Studies conducted an investigation at the facility where the affected workers were employed. The NIOSH investigation included medical testing (including pulmonary function testing, medical questionnaires and work history documentation) and industrial hygiene exposure measurements (including grab samples, use of direct reading instruments, and full shift samples). NIOSH determined that workers at this plant had > 2 times the expected rates of chronic cough, shortness of breath, asthma, and chronic bronchitis, and nonsmokers had > 10 times the expected prevalence of airway obstruction (CDC 2007; Kreiss et al. 2002). A strong association was found between this excess of lung disease, including bronchiolitis obliterans, and airborne exposures to butter-flavoring chemicals in the facility. Diacetyl was the most prominent chemical in the butter flavorings. Two other flavoring compounds of interest, acetoin and 2,3-pentanedione, were present in significant amounts or not sampled, respectively. Workers in the area where diacetyl-containing butter flavoring was added into heated mixing vats were exposed to volatilized flavor chemicals, and a significant, positive dose response relationship was identified (CDC 2007; Kreiss et al. 2002). A follow-up investigation at six other microwave popcorn manufacturing facilities found that, in five of six plants, mixers of butter flavoring and packaging-area employees working near tanks of heated oil, with exposure to diacteyl as low as 0.2 ppm, had fixed airway obstruction consistent with bronchiolitis obliterans (Kanwal et al. 2006). Based on its occurrence in microwave popcorn manufacturing plants, bronchiolitis obliterans (and some related respiratory diseases of the small airways) became commonly known as “popcorn lung.” The findings of adverse health effects in workers at microwave popcorn plants prompted additional investigations. The CDC identified seven additional cases of bronchiolitis obliterans in workers at a flavoring manufacturing company (CDC 2007).

Diacetyl is contained in a variety of flavors in addition to butter flavor (Table 1) (OSHA 2010), and its use is not limited to microwave popcorn facilities or food flavoring production facilities. Diacetyl, 2,3-pentanedione (a structurally related replacement for diacetyl), and acetoin are used in the manufacture of many other foods for a wide range of flavors beyond butter flavorings (e.g., caramel, butterscotch, pina colada, strawberry). Many of these same flavors are common in e-cigarette flavor cartridges, and are often sold with names that we consider to be potentially appealing to children, teenagers, and young adults: Cupcake, Fruit Squirts, Waikiki Watermelon, Cotton Candy, Tutti Frutti, Double Apple Hookah, Blue Water Punch, Oatmeal Cookie, and Alien Blood. Further, e-cigarettes use a battery-driven nicotine delivery system in which an atomizer produces an aerosol (and vapors of evaporated liquids) through the heating of e-cigarette liquids contained in replaceable cartridges or re-fillable wells (Burstyn 2014; Jensen et al. 2015).

**Table 1:** Flavors that contain diacetyl according to OSHA (OSHA 2010).

Flavor type	Flavors in this group
Dairy flavorings	Butter, cheese, cream cheese, cheesecake, milk, yogurt, ice cream, egg, ranch dressing, sour cream, buttermilk
Brown flavorings	Butterscotch, caramel, vanilla coffee, tea, toffee, chocolate, cocoa, cocoa butter, maple, brown sugar, marshmallow, peanut butter, praline, hazelnut, other nut flavors
Fruit flavorings	Strawberry, cranberry, raspberry, boysenberry, other berry flavors, fruit flavors—nearly any kind (e.g., banana, apple, grape, pear), cider, tomato
Alcohol flavorings	Brandy, rum, whisky, tequila, pina colada
Miscellaneous flavorings	Nutmeg, honey, graham cracker, vinegar, meat flavors

The heating, vaporization, and subsequent inhalation of these flavoring chemicals in e-cigarettes makes an exposure pathway for these flavorings that has significant similarities to those of the workers at the microwave popcorn facilities. In microwave popcorn manufacturing, flavorings, salt, and colorants are added to heated soybean oil (57–60°C). Kullman et al. (2005) reported that aerosols and flavoring ingredient vapors were found in these mixing rooms. The aerosol found to have a combustible fraction that ranged from 70% to 85% by weight (average 79%) and a noncombustible fraction of 21%. The aerosol was identified as salt particles and oil-coated salt particles, and much of the aerosol was of respirable size. The mixing rooms were where the highest air concentration of flavorings was found (Kullman et al. 2005).

Given the widespread use of these food flavors across many industries and the knowledge that specific chemicals/artificial flavors were developed to mimic certain natural flavors commonly used in e-cigarettes, we hypothesized that these compounds are likely used in the manufacturing of flavored e-cigarettes. We sought to expand the state of knowledge on flavoring chemicals in e-cigarettes with a particular focus on e-cigarettes sold by the largest cigarette companies and also those flavors that we deem would be appealing to children, teenagers, and young adults.

## Methods

### E-Cigarette Selection

A convenience sample of 51 e-cigarette flavors was selected for use in this study. Electronic cigarette cartridges, liquids, and their associated devices and batteries were purchased online and in retail locations. We evaluated 51 flavors, including all available flavors from three large cigarette companies (Brands A, B, and C, with 2, 2, and 7 flavors, respectively); 5 flavors from a large independent e-cigarette company (Brand D); and 24 additional flavors from three e-cigarette distributors (Brands E, F, and G; 10, 8, and 6 flavors, respectively) that we selected based on their potential appeal to children, teenagers, and young adults (Table 2). In addition, we evaluated 11 e-liquid flavors that are inserted into a cartomizer (disposable cartridge and atomizer system) (Brands H and I; 6 and 5 flavors, respectively).

**Table 2:** Estimated mass of flavoring chemicals in e-cigarettes (μg/e-cigarette).

Flavor	Brand	Flavor type	Diacetyl (2,3-butanedione)	2,3-Pentanedione	Acetoin
Classic	A	Tobacco	3.9	1.0	37.5
Classic	A	Tobacco	< LOD	< LOD	< LOD
Menthol	A	Other	< LOD	< LOD	< LOD
Menthol	B	Other	< LOD	< LOD	< LOD
Original	B	Tobacco	< LOD	< LOD	< LOD
Cherry Crush	C	Fruit	< LOQ	< LOD	9.0
Cherry Crush	C	Fruit	14.7	3.4	165.6
Classic	C	Tobacco	< LOQ	0.8	18.1
Java Jolt	C	Brown	21.5	7.4	212
Menthol	C	Other	8.3	2.7	18.3
Peach Schnapps	C	Cocktail	238.9	64.4	529.2
Pina Colada	C	Cocktail	27.0	7.1	45.5
Pina Colada	C	Cocktail	1.6	< LOD	130
Pina Colada	C	Cocktail	< LOD	< LOD	< LOD
Pina Colada	C	Cocktail	< LOD	< LOD	16.5
Vanilla	C	Brown	< LOD	0.9	< LOD
Bold	D	Tobacco	5.9	< LOD	39.8
Gold	D	Tobacco	0.6	< LOD	7.0
Menthol	D	Other	4.9	< LOQ	9.6
Pomegranate	D	Fruit	< LOD	0.2	11.9
Pomegranate	D	Fruit	6.9	< LOD	41.4
Vanilla Bean	D	Brown	6.7	< LOD	13.1
Bad Apple	E	Fruit	6.0	< LOD	< LOQ
Banana	E	Fruit	< LOD	< LOD	< LOQ
Cin	E	Other	38.4	23.4	< LOQ
Iced Berry	E	Fruit	6.6	< LOD	33.4
Iced Berry	E	Fruit	2.6	< LOD	17.3
Just Guava	E	Fruit	< LOQ	< LOD	7.3
Kick!	E	Brown	20.0	< LOD	19.1
Lime and Coconut	E	Fruit	10.3	< LOD	77.9
Peach Pit	E	Fruit	< LOD	< LOD	6.1
Snap!	E	Brown	10.9	3.4	88.2
Strawberry	E	Fruit	< LOQ	< LOD	5.2
Cherry	F	Fruit	4.2	< LOD	35.6
Double Apple Hookah	F	Fruit	21.1	2.3	193.5
Franks Lemon Lime	F	Fruit	4.2	1.1	47.3
Grape Hookah	F	Fruit	1.5	1.6	27.9
Orange Mint	F	Fruit	1.1	1.5	27.9
Peach	F	Fruit	8.3	< LOD	117.5
Pina Colada	F	Cocktail	11.6	0.7	55.8
Watermelon	F	Fruit	13.3	1.4	224.3
Watermelon	F	Fruit	7.4	< LOD	72.5
Bluewater Punch	G	Fruit	< LOD	< LOD	3.8
Cherry Lava	G	Fruit	< LOD	< LOD	5.6
CooCoo Coconut	G	Brown	1.0	< LOD	19.5
Milk Chocolate	G	Dairy	< LOD	< LOD	9.7
Pineapple Punch	G	Fruit	< LOD	< LOD	10.4
Waikiki Watermelon	G	Fruit	4.4	< LOQ	2.1
Alien Blood	H	Fruit	0.4	< LOD	19.4
Carmel Popcorn	H	Brown	0.3	< LOD	1.5
Cupcake	H	Brown	0.3	4.6	1.3
Energy Drink	H	Other	< LOD	< LOD	12.2
Fruit Squirts	H	Fruit	0.9	< LOD	114.4
Oatmeal Cookie	H	Other	2.2	4.2	26.1
Bubble Gum	I	Other	< LOD	< LOD	< LOD
Cheesecake	I	Dairy	0.9	< LOD	< LOD
Cola	I	Brown	< LOQ	0.2	3.7
Cotton Candy	I	Fruit	0.8	< LOD	8
Tutti Frutti	I	Fruit	9.3	0.8	24.7
< LOQ: detected by the laboratory above the laboratory limit of detection (LOD) but less than the limit of quantification (LOQ); LOQ by batch 1 and batch 2 (diacetyl: 2.3 μg, 0.19 μg; 2,3-pentanedione: 0.07 μg, 0.38 μg; acetoin: 1.08 μg, 3.2 μg). < LOD: not detected above the laboratory limit of detection (LOD), 0.05 μg.					

The emissions from the e-cigarette are composed of an aerosol and flavor/solvent vapors. The aerosol and vapors are released after contact of the flavoring solution with the heater coil in the atomizer/cartomizer. In this study we used OSHA method 1012 for sampling of three flavoring chemicals (OSHA 2008). The sampling media consists of a glass wool plug and glass fiber filter (GFF) in front of a dried silica bed. During the development of OSHA method 1012, the effectiveness of the silica gel tubes for capturing diacetyl and acetoin was examined. In part of that assessment, samples were taken with a PVC (polyvinyl chloride) filter with a powder on its surface that contained a known amount of the flavoring chemicals. The PVC filter was placed in series (before the silica gel tubes) and samples were taken. Both the filter and silica gel tubes were analyzed for the flavoring chemicals. OSHA reported that between 94.4% and 99.7% of the flavorings that were present in the powder were recovered from the silica gel tubes, not the filter—the majority of the flavoring chemicals were stripped away and captured on the silica gel (OSHA 2008).

### Sampling Protocol

**Figure 1:**
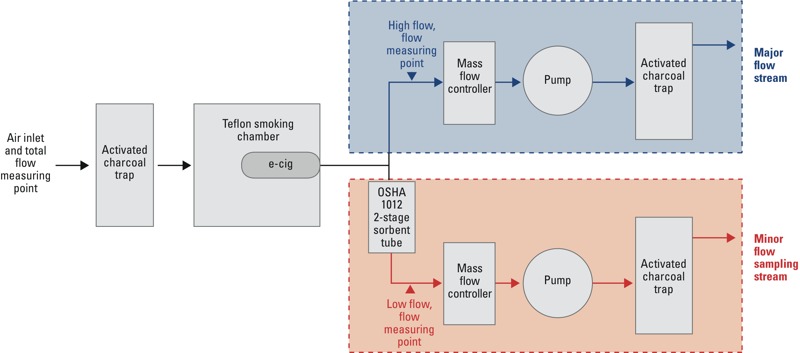
Schematic of sampling apparatus.

The goal of the sampling protocol was to estimate the total mass of diacetyl, 2,3-pentanedione, and acetoin emitted from each cartridge. Each e-cigarette was inserted into a sealed chamber attached to a laboratory-built device that drew air through the e-cigarette for 8 sec at a time, with a resting period of 15 or 30 sec between each draw (Figure 1). Eight seconds was chosen to make certain that each draw had adequate time for the entire contents to be forced out of the smoking device and through the sampling media. The draws were automated using a Pneucleus Technologies LLC MediFlo Mass Flow Controller. Air from the chamber was split into high and low flows to meet the lower-flow sampling requirements for OSHA method 1012 (OSHA 2008). The low flow (target, 200 mL/min) was optimized to carry the emissions of the e-cigarette through the sampling media (two SKC silica gel sorbent tubes containing 600 mg of specially washed and baked silica gel connected in series). The higher flow path was filtered and discharged. The total flow was set to the minimum needed to initiate a draw from the automatic e-cigarette and was measured at the beginning and end of each sample to determine the ratio between the high and low flows. The samples were collected until the e-cigarette cartridges or cartomizers were exhausted, determined by the lack of visible emissions in the chamber.

The samples from the lower flow portion of the sampling system were analyzed for diacetyl, 2,3-pentanedione, and acetoin using OSHA method 1012 (OSHA 2008). To determine the total concentration emitted, the reported values from the laboratory were adjusted using the corresponding ratio of low flow to total flow for each sample. For example, if the volume of air passing through the sampling media was 10% of total flow, the mass reported by the laboratory was multiplied by 10 to estimate the total chemical mass emitted from the e-cigarette cartridge. For the first batch of samples, the 0.12-L volume chamber was passively purged for approximately 10 min between sampling different e-cigarettes by opening the chamber. For the second batch of samples, we included a 10-min active purge of the chamber where the pumps were turned on and fresh air was drawn into the chamber. Quality assurance/quality control samples were collected for each batch.

### Quality Assurance/Quality Control

Seven blank samples (> 10% of total sample size) were collected using the same procedure outlined in the previous section but without an e-cigarette in the chamber. The same ratio adjustment process was conducted using the ratio of the low and high flow rates to obtain the total chemical mass of chemical in the blanks, if any. Values for all three chemicals were < LOD (limit of detection) in four of the seven field blanks, one had detectable levels of diacetyl and acetoin (1.2 and 10.7 μg/e-cigarette, respectively), one had detectable levels of 2,3-pentanedione and acetoin (0.4 and 9.2 μg/e-cigarette, respectively), and one had detectable acetoin only (1 μg/e-cigarette). Once the blanks were adjusted for flow, we performed a blank correction by batch according to the following procedure. Blank samples were averaged by batch before blank correction, and values below the laboratory LOD were imputed with a value of one-half the detection limit before averaging. We calculated a limit of quantification (LOQ) for our procedure that was higher than the laboratory-reported LOD (0.05 μg/sample) using three times the standard deviation of the field blank samples. After blank correction, primary samples were compared to this LOQ by batch 1 and batch 2 (diacetyl: 2.3 μg, 0.19 μg; 2,3-pentanedione: 0.07 μg, 0.38 μg; acetoin: 1.08 μg, 3.2 μg). If the blank-corrected mass was above the LOQ, the chemical was labeled “detected” and the value reported, and if the blank-corrected mass was not above the LOQ but still detected, we reported the value as “< LOQ.” If the sample was reported as not detected by the lab, we report the value as “< LOD.” We re-sampled several of the same flavors from the same package (ie, testing two e-cigarette cartridges from the same pack). These replicate samples were collected for six flavors: Brand C Pina Colada (3 replicates), Brand C Cherry Crush, Brand D Pomegranate, Brand E Iced Berry, Brand F Watermelon, and Brand A Classic. The root mean square error (RMSE) for replicate samples ranged from 2.9 μg/e-cigarette to 98.4 μg/e-cigarette.

All samples were analyzed by ALS Laboratories in Salt Lake City, Utah, a laboratory accredited by the AIHA (American Industrial Hygiene Association) Laboratory Accreditation Program for Industrial Hygiene. To check the integrity of the calibration curve, a separate initial calibration verification (ICV) standard was introduced at the mid-range level of the curve. A separate stock solution was used to generate a liquid calibration standard (LCS; 89.9% recovery) and liquid calibration standard duplicate (LSCD; 89.9% recovery) as an overall accuracy and precision check. A reagent blank was prepared and run along with the samples to ensure that the laboratory did not introduce any contamination that would affect the analyte of interest recovery. For the three analytes in this study, the reagent blanks reported levels were all less than the laboratory reporting limit of 0.05 μg per sample.

### Statistical Analysis

We grouped the samples using the product names and descriptions on the distributors’ websites into the following flavoring categories based on OSHA’s categories (Table 1): dairy, brown, fruit, and cocktail. Additional categories were created for tobacco-flavored e-cigarettes and flavors that fell into any other categories. Distributions of the mass of each of the three chemicals were compared according to flavor type using two sample *t*-tests and boxplots (R version 3.0.0; R Core Team 2015). When summarizing distributions, we substituted one half the value of the LOD as the mass for samples < LOD or < LOQ.

## Results

The total mass per e-cigarette (micrograms per e-cigarette) of the flavoring chemicals diacetyl, 2,3-pentanedione, and acetoin are presented in Table 2. Diacetyl was above the LOD in 39 of the 51 flavors tested, ranging from < LOQ to 239 μg/e-cigarette. 2,3-Pentanedione and acetoin were detected in 23 and 46 of the 51 flavors tested at concentrations up to 64 and 529 μg/e-cigarette, respectively.

At least one of the flavoring chemicals was detected in 47 of the 51 unique flavors tested (92%). This includes several e-cigarette flavors that are not candy or fruit flavored, such as “classic” and “menthol.” Diacetyl and 2,3-pentanedione were detected simultaneously in 21 unique flavors, suggesting that 2,3-pentanedione may not be only a replacement for diacetyl but is often used in conjunction with diacetyl in e-cigarettes. Similarly, 2,3-pentanedione and acetoin were also detected simultaneously in 22 flavors. Diacetyl and acetoin were simultaneously detected in an even greater number of flavors (*n* = 38).

**Figure 2:**
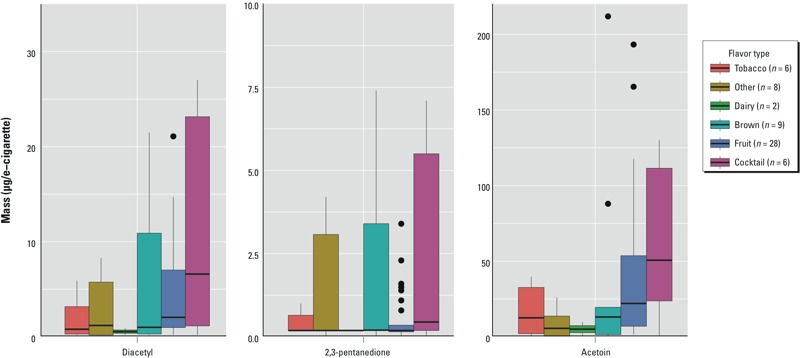
Boxplots showing the median (horizontal line in box), interquartile range (shaded box), and 1.5 times the interquartile range (vertical lines) of e-cigarette sample masses, including replicates, by flavor type for diacetyl, 2,3-pentanedione, and acetoin. Samples outside 1.5 times the interquartile range are shown as dots. The two highest concentrations for each chemical are not shown.

Figure 2 depicts the distributions of the chemical masses of the e-cigarette samples, including replicates, according to flavor type. The three compounds were detected in all flavor types, except for 2,3-pentanedione in dairy-flavored e-cigarettes, which only had two samples. The median masses of the flavor types did not have a consistent ranking from one chemical to the next. For example, tobacco flavors had the second to lowest median mass for diacetyl compared with the fourth lowest for acetoin. The cocktail-flavored e-cigarettes had the highest median masses and largest range for all three compounds, but none of the differences in the mean masses of each flavor type were statistically significant, using the Bonferroni correction for multiple comparisons.

## Discussion

Diacetyl—a flavoring compound associated with the development of “popcorn lung” in workers after inhalation exposure—was detected in 39 of the 51 flavored e-cigarettes tested in this study, including flavors that have particular appeal to children, teenagers, and young adults. Forty-seven of the 51 flavors tested in our study had at least one of the three flavoring compounds detected (diacetyl, 2,3-pentandedione, acetoin). These compounds were ubiquitous among flavor types: “tobacco” and “menthol” flavored e-cigarettes contained diacetyl despite not being listed on OSHA’s list of flavors that likely contain diacetyl (Table 1).

The health concerns related to inhaling diacetyl and other flavoring chemicals are now well recognized by OSHA and the flavoring industry. OSHA established a National Emphasis Program (NEP) in 2007 focused on respiratory disease in workers at microwave popcorn–processing facilities, and a NEP in 2009 focused on “Facilities that Manufacture Food Flavorings Containing Diacetyl” (OSHA 2007, 2009). The Flavoring and Extract Manufacturers Association of the United States released a report in April 2012 on respiratory health and safety in the food-manufacturing workplace that highlighted the potential risks associated with inhaling diacetyl and a long list of other food flavoring chemicals (FEMA 2012). FEMA recommends the following warning for “Any compounded flavors (liquid, dry or powdered) containing any flavoring substances listed in Table 1 in any concentrations if the compounded flavor or any of its individual flavoring substances will be heated during processing.” [The Table 1 referenced is specific to diacetyl.]

WARNING–This flavor may pose an inhalation hazard if improperly handled. Please contact your workplace safety officer before opening and handling, and read the MSDS. Handling of this flavor that results in inhalation of fumes, especially if the flavor is heated, may cause severe adverse health effects.

Unlike these efforts by OSHA and the flavor industry to raise awareness of the hazards associated with inhaling flavoring chemicals, our review of the websites and packaging for the flavored e-cigarette brands in our study did not identify any similar notifications regarding diacetyl specifically or flavorings generally. Two companies explicitly stated that their products do not contain diacetyl in written communication, yet in our testing we did find diacetyl in their product.

Rules for labeling do not currently exist for e-cigarettes, because—unlike tobacco products, which are regulated by the FDA under authority of the Family Smoking Prevention and Tobacco Control Act (2009), a statute that authorizes the FDA to require warning labels on packages and advertisements and bans flavored cigarettes—e-cigarettes are not currently regulated. However, this may be changing. In 2014, the FDA issued a proposed rule that seeks to expand the legal definition of tobacco products to include e-cigarettes and other nicotine-containing products (FDA 2014). If finalized, the rule may include minimum age and identification requirements and proposed addictiveness warnings. Specifically related to the research presented here, and our opinion that many flavors are appealing to youth, the FDA (2014) states that “some tobacco products, such as e-cigarettes and certain cigars, are being marketed with characterizing flavors, and that these flavors can be especially attractive to youth.” The FDA then acknowledges that the existing Family Smoking Prevention and Tobacco Control Act of 2009 prohibiting flavors currently applies only to cigarettes, not e-cigarettes, and they are seeking additional information regarding the effects e-cigarettes have on public health. The data presented in this manuscript on the presence of flavoring chemicals in e-cigarettes that have been previously associated with severe respiratory disease are a step toward addressing this information gap.

As a result of the toxicological and epidemiological studies by NIOSH, inhalation exposure limits for adult workers have been established for several food-flavoring compounds, including diacetyl and its structurally similar replacement, 2,3-pentanedione (Table 3). However, there are no health-based standards for diacetyl inhalation for the general public, and no standards for children. We agree with a recent response to an article by NIOSH investigators (Hubbs et al. 2015) and an advisory released by FEMA (2015) that there are important considerations in interpreting analyses that use occupational health limits for estimating risk for e-cigarette smokers. First, these occupational health limits are set for healthy workers, not the general population, and e-cigarette users are not exclusively workers. Second, the U.S. regulatory agencies accept greater risk for workers than for the general population. For example, “acceptable” risk for workers is generally 1 in 1,000 to 1 in 10,000 risk of an adverse event (Hubbs et al. 2015), whereas the U.S. EPA (Environmental Protection Agency) uses 1 in 100,000 to 1 in 1,000,000 as “acceptable” for the general population (Castorina and Woodruff 2003). By applying occupational health limits to the general population of flavored e-cigarette smokers, we would thus be accepting a higher risk than typical. Third, the occupational limits are based on an 8-hr period, 5 days per week, and come with the assumption that a worker will have 16 hr of recovery time between shifts, and 2-day recovery on the weekend, which is not applicable to e-cigarette users. Fourth, these exposure limits are for adults, not children, who on average have a smaller body weight compared with typical adult workers, resulting in a greater overall dose per e-cigarette for children and adolescents. Fifth, we do not know whether the dose–response relationships observed for workers would be similar for children, who can be more susceptible to some environmental exposures. Last, the occupational exposure limits are not “bright lines”; values below the limit should not automatically be interpreted as “safe.” In fact, there is guidance for interpreting values below the occupational health limits. The AIHA first uses the upper confidence limit of the 95th percentile exposure value when comparing exposure measurements to an occupational limit, not just a point estimate or mean. They then use a “control banding” to establish where the exposure fits within five AIHA exposure control categories (Ignacio and Bullock 2006). Even when the 95th percentile exposure estimate is 10% of the occupational limit, at a minimum hazard communication is a typical control response to the exposure (Hewett et al. 2006). At 50% of the occupational limit, additional controls are also typical, including exposure surveillance, medical surveillance, and work practice evaluation (Hewett et al. 2006).

**Table 3:** Occupational exposure guidelines in parts per million.

Agency	Averaging time	2,3-Pentanedione	Diacetyl	Acetoin	Reference
OSHA PEL	8-hr	NA	NA	NA	OSHA 2015
	16-hr*	NA	NA	NA
NIOSH REL	8-hr	0.0093^*a*^	0.005^*a*^	NA (10-hr)	NIOSH 2011
	16-hr*	0.0023	0.00125	NA
NIOSH STEL	15-min ceiling	0.031^*a*^	0.025^*a*^	NA	NIOSH 2011
ACGIH TLV	8-hr	NA	0.01	NA	ACGIH 2014
	16-hr*	NA	0.0025	NA
ACGIH STEL	15 min	NA	0.02	NA	ACGIH 2014
Abbreviations: NA, not available; PEL, permissable exposure limit; REL, recommended exposure limit; STEL, short-term exposure limit; TLV, threshold limit value. ^***a***^Draft occupational exposure limit (ppm). *Adjusted OEL (occupational exposure limit) using Brief and Scala method.					

### Strengths and Limitations

One major goal of our study was to determine whether diacetyl and other flavoring compounds were present in the vapors released from flavored e-cigarettes. Because of the vast number of flavored e-cigarettes currently on the market, our convenience sample of 51 flavors means that the extent to which are results are generalizable to the entire population of e-cigarette flavors is simply unknown; we did, however, detect at least one flavoring compound in 47 of the 51 flavors tested, suggesting the need to rapidly determine whether this high prevalence found in our study is consistent across the many thousands of flavors being sold. Our method for determining when the e-cigarette was fully spent relied on a visual determination of emissions of the e-cigarette in the chamber. It is possible that our samples did not fully reflect the total chemical content in the e-cigarettes if liquid remained in the e-cigarette at the time our sampler was turned off, causing an underestimate of chemical content. This method may explain the variability in replicate samples, as well as variable chemical doses in e-cigarettes of the same type. Another approach to determine total content would be to directly analyze the chemical content in the liquid contained in flavored e-cigarettes, but this does not permit analysis of the vapor. Also, our blank correction procedure used an imputed value for blank samples that were less than the limit of detection. Typically, blank correction would not be performed when blank samples do not have detectable levels of a chemical. However, to be consistent in how we handled blanks that did have detectable levels across the different batches, we decided to uniformly blank-correct, including the incorporation of nondetected blank samples (imputed, as previously described). This also would lead to an underestimate of chemical concentrations in the vapor. Last, our samples also showed within-flavor variability, as evidenced by the RMSE for replicate samples. This variability could be attributable to our method or variability in chemical content in flavored e-cigarettes; Cheng (2014) reported a high degree of variability in nicotine content within e-cigarettes of the same brand. Given these limitations, we urge caution in interpreting samples with values below the limit of quantification but above the limit of detection as being “diacetyl-free.”

Strengths of our approach include measuring actual concentrations of these three flavoring chemicals in the vapor of e-cigarettes using repeatable and validated sampling and analytical methods, and methodological decisions that gave us confidence in reporting the presence of diacetyl in e-cigarettes. Future studies should refine these and other methods to further quantify the amount of flavoring chemicals in e-cigarettes, and the prevalence of diacetyl and alternative flavoring chemicals in a wider range of samples. Last, studies need to be performed to assess potential differences between the particulate and vapor contribution to exposure and test other environmental conditions, including variability in humidity, differences in smoker draw time/pressures, and different designs of the vaporization systems. For example, Zhang et al. (2013) showed that puffs generated smaller peak particle sizes than did drawing at a constant rate, which has implications for where inhaled particles will penetrate in the lungs. A standardized protocol for evaluating emissions (particulate and vapors) of e-cigarettes would facilitate interpretation of study results.

## Conclusion

Our findings confirm the presence of diacetyl and other flavoring chemicals in flavored e-cigarettes. Because of the associations between diacetyl and bronchiolitis obliterans and other severe respiratory diseases among workers inhaling heated vapors containing diacetyl, urgent action is recommended to further evaluate the extent of this new exposure to diacetyl and related flavoring compounds in e-cigarettes.
